# Maintaining *Plasmodium falciparum* gametocyte infectivity during blood collection and transport for mosquito feeding assays in the field

**DOI:** 10.1186/s12936-021-03725-y

**Published:** 2021-04-20

**Authors:** Harouna M. Soumare, Wamdaogo Moussa Guelbeogo, Marga van de Vegte-Bolmer, Geert-Jan van Gemert, Zongo Soumanaba, Alphonse Ouedraogo, Maurice S. Ouattara, Ahmad Abdullahi, Lamin Jadama, Muhammed M. Camara, Pa Modou Gaye, Michael Mendy, Nwakanma Davis, Alfred B. Tiono, Umberto D’Alessandro, Chris Drakeley, Teun Bousema, Marta Moreno, Katharine A. Collins

**Affiliations:** 1 Medical Research Council Unit, The Gambia at the London School of Hygiene and Tropical Medicine, Banjul, The Gambia; 2 Centre National de Recherche Et de Formation Sur Le Paludisme, Ouagadougou, Burkina Faso; 3 Department of Medical Microbiology, Radboud University Medical Center, Nijmegen, The Netherlands; 4 Department of Biology of Infection, London School of Hygiene and Tropical Medicine, Faculty of Infectious and Tropical Diseases, London, UK

**Keywords:** Gametocyte, Anopheles, Mosquitoes, Malaria, Activation, Falciparum, Transmission

## Abstract

**Background:**

Mosquito feeding assays using venous blood are commonly used for evaluating the transmission potential of malaria infected individuals. To improve the accuracy of these assays, care must be taken to prevent premature activation or inactivation of gametocytes before they are fed to mosquitoes. This can be challenging in the field where infected individuals and insectary facilities are sometimes very far apart. In this study, a simple, reliable, field applicable method is presented for storage and transport of gametocyte infected blood using a thermos flask.

**Methods:**

The optimal storage conditions for maintaining the transmissibility of gametocytes were determined initially using cultured *Plasmodium falciparum* gametocytes in standard membrane feeding assays (SMFAs). The impact of both the internal thermos water temperature (35.5 to 37.8 °C), and the external environmental temperature (room temperature to 42 °C) during long-term (4 h) storage, and the impact of short-term (15 min) temperature changes (room temp to 40 °C) during membrane feeding assays was assessed. The optimal conditions were then evaluated in direct membrane feeding assays (DMFAs) in Burkina Faso and The Gambia where blood from naturally-infected gametocyte carriers was offered to mosquitoes immediately and after storage in thermos flasks.

**Results:**

Using cultured gametocytes in SMFAs it was determined that an internal thermos water temperature of 35.5 °C and storage of the thermos flask between RT (~ 21.3 °C) and 32 °C was optimal for maintaining transmissibility of gametocytes for 4 h. Short-term storage of the gametocyte infected blood for 15 min at temperatures up to 40 °C (range: RT, 30 °C, 38 °C and 40 °C) did not negatively affect gametocyte infectivity. Using samples from natural gametocyte carriers (47 from Burkina Faso and 16 from The Gambia), the prevalence of infected mosquitoes and the intensity of oocyst infection was maintained when gametocyte infected blood was stored in a thermos flask in water at 35.5 °C for up to 4 h.

**Conclusions:**

This study determines the optimal long-term (4 h) storage temperature for gametocyte infected blood and the external environment temperature range within which gametocyte infectivity is unaffected. This will improve the accuracy, reproducibility, and utility of DMFAs in the field, and permit reliable comparative assessments of malaria transmission epidemiology in different settings.

**Supplementary Information:**

The online version contains supplementary material available at 10.1186/s12936-021-03725-y.

## Background

In the past decade, remarkable reductions in malaria burden have been achieved largely through widespread access to and use of artemisinin-based combination therapy and insecticide-treated bed nets [1]. However, since 2015, progress has stalled [2] and elimination is unlikely to be achieved with these conventional methods alone in most settings in Africa [3]. One of the biggest challenges to malaria control and elimination is effectively interrupting the efficient process of malaria transmission. New tools or implementation strategies will likely be required that specifically aim to reduce malaria transmission [4]. In order to effectively target transmission reduction and develop and implement transmission-blocking interventions, a thorough understanding of the dynamics and epidemiology of malaria transmission in different settings is needed.

The transmission of malaria depends on the presence of mature sexual stage parasites (gametocytes) in the peripheral blood. During a blood meal, *Anopheles* mosquitoes must imbibe at least one male and one female gametocyte to become infected, but other host or parasite factors may also contribute to this, such a transmission reducing immunity. To understand how likely it is that individuals contribute to onward transmission, assays are needed for accurate assessment of gametocyte infectivity to mosquitoes. This is commonly measured by direct membrane feeding assays (DMFAs) where venous blood is fed to mosquitoes, or direct skin feeding assays, where mosquitoes feed directly on the skin of the volunteer [5–11]. In DMFAs, venous blood collected from malaria infected individuals is offered to female *Anopheles* mosquitoes using water-jacketed glass feeders. These feeders are connected to a circulating water bath to maintain gametocyte temperature during feeding. Maintaining gametocyte temperature is important since a drop in temperature may result in gametocyte activation [12] while high temperatures (~ 40 °C or higher) may inactivate gametocytes and gametes [13–16].

Blood drawn for DMFAs must therefore be fed to mosquitoes as quickly as possible after collection to eliminate any detrimental effect of temperature change [17]. This can be logistically challenging in the field where participants in DMFA studies may be recruited far from the insectary facilities, resulting in either lengthy travel for volunteers, or restricting the available population for infectivity assessment to those near the facilities. To overcome these limitations, a method to store and transport gametocyte infected blood from the field to the laboratory and prevent premature activation or inactivation of gametocytes before mosquito feeding is needed.

In this study, the temperature range for storage of gametocyte infected blood to maintain gametocyte infectivity is determined, and a simple, cheap, field applicable method for collecting and transporting blood is presented.

## Methods

### Mosquito rearing

*Anopheles stephensi* Sind-Kasur Nijmegen strain [18] (Radboudumc) were reared at 30 °C, and *Anopheles coluzzii* (Burkina Faso and The Gambia) were reared at 27 °C. All were kept at ~ 70–80% relative humidity (RH) on a 12-h day/night cycle and were fed on 5–10% glucose. Female mosquitoes between 2 and 6 days post emergence were used for all mosquito feeding assays. After feeding on gametocyte infected blood, mosquitoes were stored either at 26 °C (Radboudumc) or 27 °C (Burkina Faso and The Gambia).

### Standard membrane feeding assays (SMFAs)

SMFAs were performed at Radboudumc, Nijmegen, The Netherlands, using cultured *Plasmodium falciparum* gametocytes as previously described [19]. In brief, *P. falciparum* was cultured at 37 °C in an automated incubator under a continuous gas flow of 4% CO_2_, 3% O_2_ and 93% N_2_. Gametocytes were produced from asynchronous cultures (day 0, 1% parasitaemia and 5% red blood cells), which were harvested at day 15. Gametocyte infected blood meals were prepared by adding 900 µl mature stage 5 gametocyte culture to 600 µl packed red blood cells, this was briefly centrifuged, the supernatant removed and 600 µl human serum added. For the long-term storage experiments, gametocyte infected blood meals were either fed immediately to mosquitoes or were stored for 4 h in a tube in a thermos flask filled with water at a range temperatures, measured using a calibrated probe thermometer accurate to ± 0.2 °C (Traceable® Ultra digital thermometer, VWR 620-2079) before feeding to mosquitoes. The water temperatures chosen were either the temperature hypothesized to be the optimal gametocyte storage temperature (the temperature of the human body), 37 °C (range: 36.9–37.2 °C) or an arbitrarily selected lower (35.5 °C [range: 35.4–35.6 °C]) or higher (37.8 °C [range: 37.8–37.8 °C]) temperature. Thermos flasks (Stainless king food flask 470 ml, Thermos) were stored either at room temperature (RT; mean 21.3 °C, range: 20.4–22.1 °C) or in an incubator set at 32 °C (range: 31.9–32.2 °C) or 42 °C (range: 41.4–42.1 °C). These storage temperatures were selected to reflect the range of temperatures that might be experienced during thermos flask storage in a malaria endemic county. The exact thermos storage temperature and water temperatures are presented in Additional file 1: Table S1. For the short-term storage experiments, gametocyte infected blood meals were stored for 15 min at RT or in a heat block at a range of temperatures (30 °C, 35.5 °C, 38 °C, 40 °C or 42 °C) before feeding to mosquitoes. For all SMFAs, female mosquitoes were offered the infected blood meal in glass mini-feeders (300 μL) attached to a circulating water bath set at 39 °C and allowed to feed in the dark for 15 min [19]. A total of 30–60 mosquitoes in 2 or 3 cups were used for each experimental condition. Mosquitoes were maintained on 5% glucose and dissected 6–8 days after feeding and the number of developed oocysts per mosquito was determined by microscopy after 1% mercurochrome midgut staining.

### Ethical approval

The Burkina Faso study was approved by the London School of Hygiene and Tropical Medicine ethics committee (Review number: 14724), the Centre National de Recherche et de Formation sur le Paludisme institutional review board (Deliberation N° 2018/000,002/MS/SG/CNRFP/CIB) and the Ethics Committee for Health Research in Burkina Faso (Deliberation N° 2018–01-010). The Gambia study was approved by the London School of Hygiene and Tropical Medicine ethics committee (Review number:15993) and by The Gambia Government/MRC Joint Ethics Committee (Review number:1621). All participants gave informed consent before inclusion in the studies.

### Gametocyte positive blood samples

Gametocyte positive human blood samples were obtained from individuals as part of ongoing studies in Burkina Faso and The Gambia. In Burkina Faso, participants were microscopy-positive gametocyte carriers aged 10–15 years old, recruited from screening campaigns in the Saponé health and demographic surveillance system area, ~ 45 kms Southwest of Ouagadougou. In The Gambia, participants were microscopy-positive gametocyte carriers aged > 2 years, passively recruited from four health facilities (Basse Hospital, Sabi, Sotuma Sere, Gambissara) in the South Bank of the Upper River Region in The Gambia.

### Direct membrane feeding assays (DMFAs)

#### Thermos flask water temperature setup

Thermos flasks (Stainless king food flask 470 ml, Thermos) were filled (to 2 cm below the top of the flask) with water between 35.5 and 36 °C, measured with a calibrated probe thermometer (accurate to ± 0.2 °C, Traceable® Ultra digital thermometer, VWR 620-2079) and immediately sealed. Gametocyte infected blood samples were collected in Lithium heparin vacutainers and either fed to mosquitoes immediately (control) or transferred to the thermos flask immediately after phlebotomy. One thermos flask was used per blood donor to avoid repeated opening of the flask, and 2 tubes of blood were collected from each donor. Thermos flasks were used within 1 h of it being filled with water. Thermos flasks were then stored at ambient temperature in the laboratory or in an air-conditioned car (range: 25.5–29 °C) for a maximum of 4 h from the time of filling and the stored blood sample was then used for mosquito feeding.

#### Feeding procedures

For all DMFAs, female mosquitoes were offered the infected blood meal in glass mini-feeders (300 μL) attached to a circulating water bath at 37–39 °C and allowed to feed in the dark for 15–20 min. A total of 3 cups of 30 mosquitoes were used for all experimental conditions in The Gambia, and 2 cups of 60 mosquitoes were used for all experimental conditions in Burkina Faso. Mosquitoes were maintained on 5–10% glucose until 7–8 days after feeding when they were dissected and the number of developed oocysts per mosquito was determined by microscopy after 0.5% mercurochrome midgut staining [20].

### Statistical analysis

Statistical analysis was performed using GraphPad Prism (ver. 8). Mosquito infectivity in SMFAs was analysed by comparing groups with the Kruskal–Wallis test comparing each experimental condition to the control with Dunns multiple comparison test. Mosquito infectivity in DMFAs was analysed by comparing oocyst intensity (average oocysts per mosquito) or prevalence of infection (% of infected mosquitoes) by Wilcoxon matched-pairs signed rank test. Agreement between the immediate DMFAs or DMFAs with stored gametocytes was analysed using Spearmans correlation of % mosquitoes infected before and after gametocyte storage and Bland–Altman analysis to visualize and test whether systematic bias in infection rates occurred between the two methods.

## Results

### Optimal storage conditions for cultured gametocytes for SMFAs

**Fig. 1:**
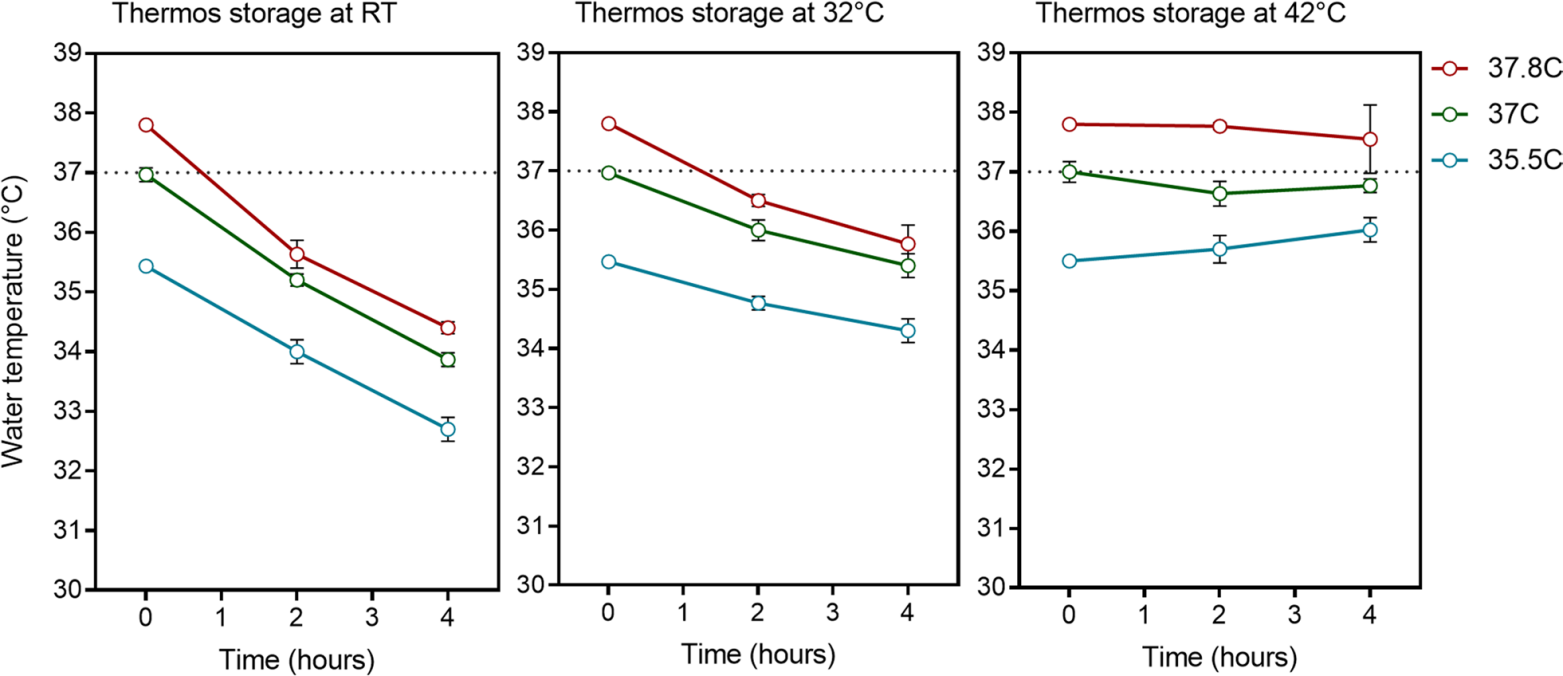
Water temperature stability in thermos flasks. Water temperature was measured with a calibrated thermometer at the start of the incubation and after 2 and 4 h of storage. Each thermos flask was opened only once during the 4-h storage to measure the temperature, and sealed again immediately after. Each condition (starting water temperature [35.5 °C, 37 °C or 38 °C] and storage temperature [RT, 32 °C or 42 °C] combination) was evaluated 3–4 times. Graphs show the mean and SD for each condition over time

To determine the optimal storage conditions for cultured stage 5 gametocytes that maintains their infectivity, SMFAs were performed where two conditions were evaluated; (i) the starting water temperature in the thermos flask and (ii) the temperature the sealed thermos flask was stored at. In a series of experiments, mosquito blood meals were prepared with mature stage 5 gametocyte cultures and either fed immediately to mosquitoes (control) or stored in a thermos flask for 4 h. The thermos flasks were filled with water at either 37.8 °C, 37 °C or 35.5 °C and after addition of the mosquito blood meal the thermos flasks were sealed and stored at either room temperature (RT), 32 °C or 42 °C. The stability of the water temperature was evaluated 2 and 4 h after initial filling of the thermos for all experiments (Fig. 1 and Additional file 1: Table S1). The impact of storing the cultured gametocytes in the thermos flask was assessed by comparing the level of transmission, as measured by the intensity of mosquito oocyst infection, in the immediate control SMFA to the SMFA performed with the stored gametocytes.

**Fig. 2:**
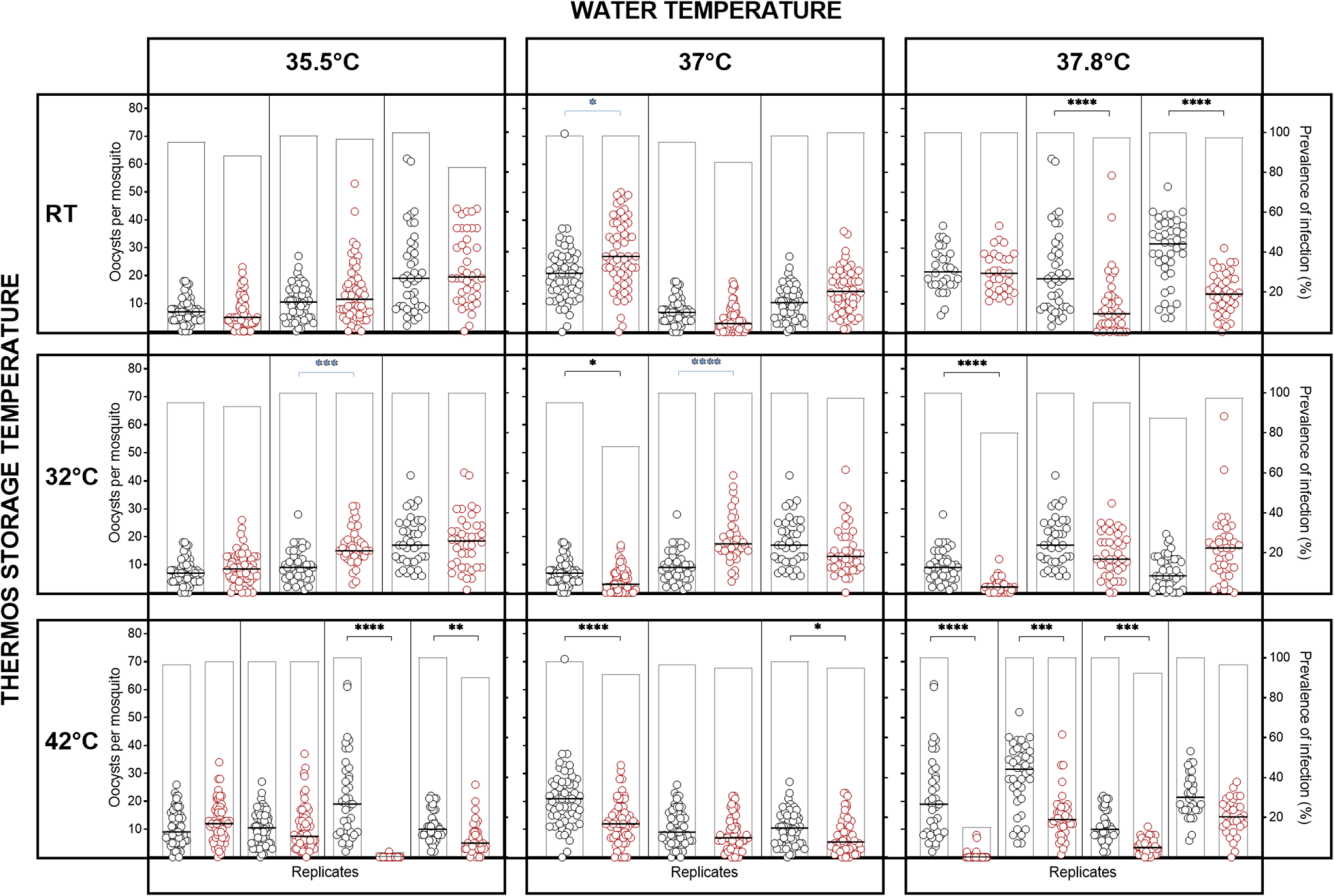
Infectivity of cultured gametocytes following thermos flask storage. Mosquito infection rate (intensity of oocyst infection) was compared when a gametocyte infected blood meal was fed to mosquitoes immediately (black circles) or stored in a thermos flask for 4 h (red circles). Two storage condition variables were evaluated; (i) the starting water temperature in the thermos flask (37.8 °C, 37 °C or 35.5 °C) and, (ii) the storage temperature of the sealed thermos flask (RT, 32 °C or 42 °C). Graphs show the intensity of infection (oocysts per mosquito) on the left Y axis (circles) and the prevalence of infection (% of mosquitoes infected) on the right Y axis (bars) using the different conditions, (lines indicate the median). A total of 30–60 mosquitoes in 2 or 3 cups were used for each experimental condition and each condition was evaluated 3 to 4 times in independent replicate experiments. Groups were compared by Kruskal–Wallis test comparing the oocyst intensity in each experimental condition to its own control with Dunns multiple comparison test. Statistical comparisons indicated in black show significant reductions, and in blue show significant increases in transmission

During the 4 h of storage, the thermos flasks were opened once to measure the temperature before being sealed again and the temperature measured a final time after 4 h. The water temperatures remained relatively stable during storage, with a greater reduction in water temperature observed when the thermos was stored at RT (mean change  −3.08 °C) compared to 32 °C (mean change  −1.59 °C) or 42 °C (mean change 0.04 °C) (Fig. 1 and Additional file 1: Table S1). There was no reduction in transmission when the gametocyte infected blood meal was stored for up to 4 h in a thermos containing water at 35.5 °C and the thermos flask was stored between RT (~ 21.3 °C) and 32 °C. However, when the thermos was stored at a higher temperature (42 °C) there was a statistically significant reduction in intensity of oocyst infection in 50% of the experiments (Fig. 2 and Table 1). When the water in the thermos was higher, at 37 °C or 37.8 °C, a statistically significant reduction in transmission was occasionally observed after gametocyte infected blood meal storage, even when the thermos was kept between RT (~ 21.3 °C) and 32 °C. This was more frequent when the water was 37.8 °C (Table 1 and Fig. 2). The percentage of mosquitoes infected in each experiment followed the same trend as intensity of infection, but was less affected by the storage conditions. These results indicate that in order to maintain infectivity of gametocytes they should be stored in a thermos filled with water that is 35.5 °C and the thermos should be stored between 21.3 °C and 32 °C. In 3 experiments there was an increase in oocyst intensity. This was only observed in 1/6 experiments using the above proposed optimal conditions.

**Table 1:** Effect of gametocyte blood meal storage on infectivity to mosquitoes

Thermos water temperature	Thermos storage temperature	Total number of mosquitoes evaluated per condition (controls)	Number of experiments with a significant reduction in infectivity after 4 h storage
37.8 °C	RT	110 (110)	2/3
	32 °C	120 (120)	1/3
	42 °C	150 (150)	3/4
37 °C	RT	180 (178)	0/3
	32 °C	140 (140)	1/3
	42 °C	180 (180)	2/3
35.5 °C	RT	160 (160)	0/3
	32 °C	140 (140)	0/3
	42 °C	200 (200)	2/4

Mosquito infection rate (intensity of oocyst infection) was compared when a gametocyte infected blood meal was fed to mosquitoes immediately or stored in a thermos flask for 4 h at a range of temperatures. A total of 30–60 mosquitoes in 2 or 3 cups were used for each experimental condition and each experiment was repeated 3 to 4 times. Groups were compared by Kruskal–Wallis comparing each experimental condition to its own control with Dunns multiple comparison test. Graphs showing the full results of the experiments are presented in Fig. 2

### Optimal membrane feeder temperature for cultured gametocytes in SMFAs

Since the optimal temperature for 4 h of gametocyte storage was determined to be 35.5 °C, lower than expected, the standard temperature used in the membrane feeders for SMFAs (37 °C) was examined to determine whether this might also have a negative effect on gametocyte transmission to mosquitoes. To evaluate this in a series of experiments, cultured stage 5 gametocyte blood meals were stored for 15 min (the standard duration of mosquito feeding in a SMFA) at either 35.5 °C (safe temperature for 4-h storage) or a range of higher and lower temperatures (RT, 30 °C, 38 °C, 40 °C and 42 °C).

**Fig. 3:**
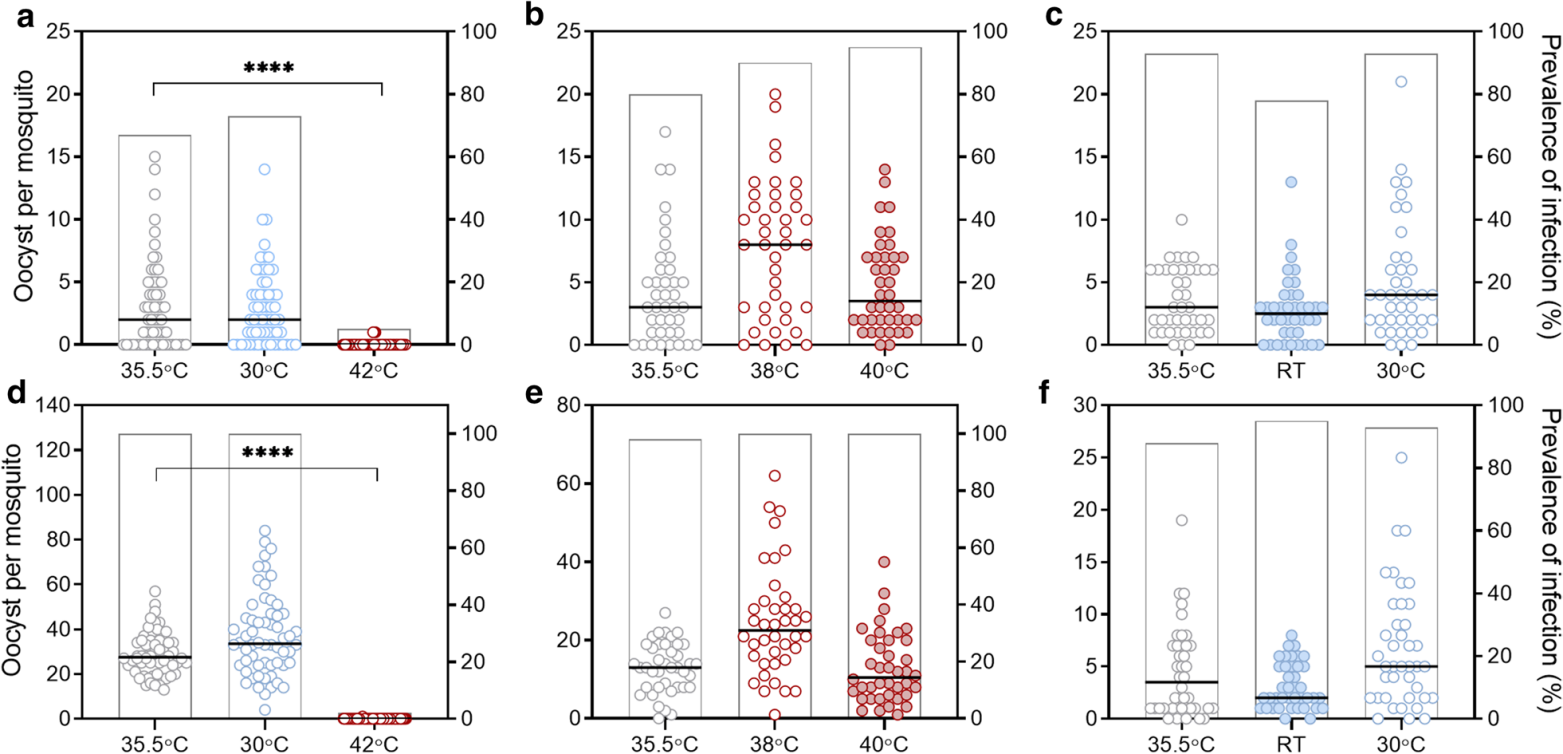
Impact of short-term gametocyte blood meal storage on gametocyte infectivity. Mosquito infection rate was evaluated from gametocyte blood-meals that were stored for 15 min at a range of temperatures (RT, 30 °C, 38 °C, 40 °C and 42 °C) and compared to the control storage at 35.5 °C (safe storage temperature for 4-h storage). A total of 30–60 mosquitoes in 2 or 3 cups were used for each experimental condition. Each experiment was performed twice, with each graph (**a**–**f**) representing the results from independent replicate experiments. Graphs show the intensity of oocyst infection (number of oocysts per mosquito) on the left Y axis (circles) and prevalence of infection (% of mosquitoes infected) on the right Y axis (bars), and the lines indicate the median. Groups were compared by Kruskal–Wallis test comparing each experimental condition to the control (35.5 °C) with Dunns multiple comparison test

Short-term storage of the gametocyte blood meal for 15 min at 42 °C resulted in significant reduction in transmission, but short-term storage at temperatures lower than this (40 °C, 38 °C, 30 °C and RT) did not have a negative effect (Fig. 3). This indicates that the temperature of the membrane feeders should not exceed 40 °C for a 15-min mosquito feed, but lower temperatures (as low as 21.3 °C) are not detrimental for this short duration.

### Validation of optimal gametocyte thermos storage conditions using natural gametocyte carriers

**Fig. 4:**
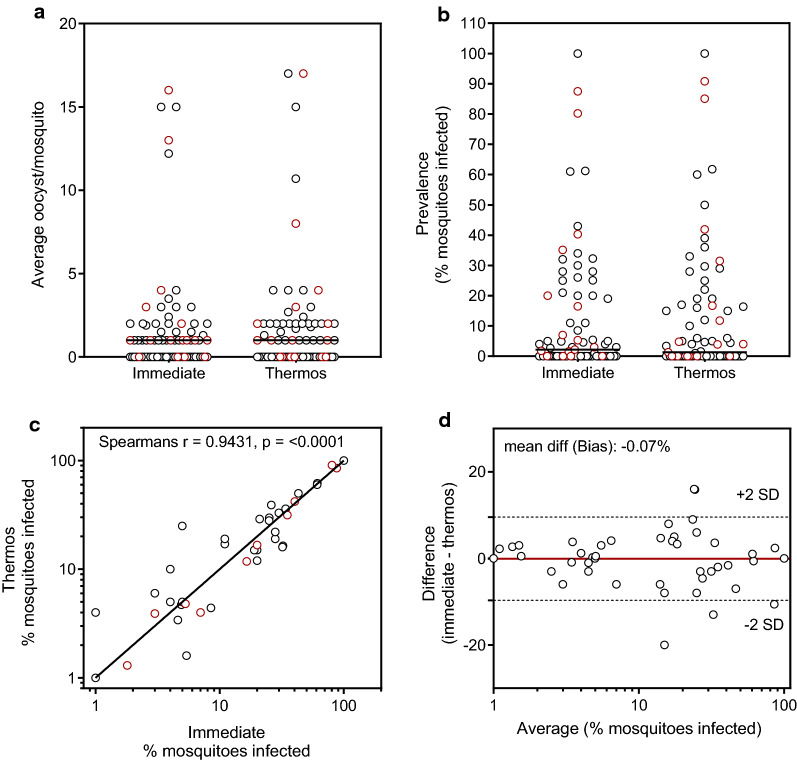
Infectivity of natural gametocytes following thermos flask storage. Gametocyte infected blood was collected from individuals in Burkina Faso (black circles, n = 47) and The Gambia (red circles, n = 16), and paired DMFAs were performed either immediately (Immediate) or following storage of the blood for 3 – 4 h in a thermos flask filled with water at 35.5 °C to 36 °C (Thermos). Mosquito infection rate was evaluated as **a** average oocysts per mosquito, or **b** prevalence of infection (% of infected mosquitoes). Lines indicate the group medians and groups compared by Wilcoxon matched-pairs signed rank test. **c** Spearmans correlation of % mosquitoes infected before and after gametocyte storage and **d** Bland–Altman analysis comparing the % mosquitoes infected using the two methods

To assess if the optimal thermos storage conditions determined using cultured gametocytes were also suitable for storage of natural gametocytes in field conditions, paired experiments (n = 47 in Burkina Faso and n = 16 in The Gambia) were performed where blood was collected from gametocyte infected individuals and either fed immediately to mosquitoes in a DMFA or stored in the thermos flask for up to 4 h before DMFA. The starting temperature of the water in the thermos flask was 35.5–36 °C, and the thermos flasks were stored at ambient lab temperature or in an air-conditioned car (range: 25.5–29 °C). The prevalence of infected mosquitoes and the intensity of mosquito infection was similar for the DMFAs performed immediately compared to the DMFAs performed after 4 h storage in a thermos flask (Fig. 4a, b). When comparing results from the two assays, there was a strong correlation (Spearman’s r = 0.9431, p < 0.0001) and good agreement using the Bland–Altman analysis (mean difference in mosquito infection rate = − 0.07%) (Fig. 4c, d).

## Discussion

In this study, a method to maintain *P. falciparum* gametocyte infectivity during blood collection in the field and transportation to the insectary for mosquito feeding assays is presented. The optimal storage conditions preventing gametocyte activation or inactivation were first assessed with cultured gametocytes and then confirmed using natural gametocyte carriers from two malaria endemic settings. The findings corroborate that temperature can play a key role in maintaining the infectivity of *P. falciparum* gametocytes. Infectivity was not affected when gametocytes were stored in thermos flasks in water at 35.5 °C for up to 4 h and the thermos flask was stored at ambient temperatures ranging from 21.3 to 32 °C.

DMFAs are more commonly used than direct skin feeding assays to assess the infectiousness of gametocyte carriers to mosquitoes, largely because they minimize the discomfort experienced by volunteers, which is particularly important when sampling young children. DMFAs may, therefore, be more readily acceptable by both local communities and ethics committees [21], especially when repeated assessments of infectivity are made. The relationship between gametocyte density and infection success in mosquitoes has been well studied, but differs slightly by settings [11, 21, 22]. These differences could plausibly be due to the different populations, different levels of malaria exposure and resulting levels of transmission-blocking immunity, different mosquito species, or parasite genotypes [8, 9, 11]. However, for reliable assessments of infectivity by DMFA it is crucial to minimize technical differences in how the assays are performed between settings [21, 22]. Here, evidence is presented that variation in blood storage temperature and duration of storage, as well as feeder temperature, could have a significant impact on transmission.

The results show that the optimal temperature for longer-term storage (4 h) is 35.5 °C. The maintenance of gametocyte infectivity at this temperature is consistent with previous studies which showed a drop in temperature of at least 5 °C from the standard 37 °C in the human body, is required to activate *P. falciparum* gametocytes [12]. Also consistent with previous data [13, 14, 16, 23], our results show that as little as 15 min (mimicking the time the gametocytes are present in the feeder) at 42 °C is sufficient to inactivate gametocytes, with transmission being almost completely prevented. Taken together, these data suggest that the temperature in the feeders should not exceed 40 °C during DMFAs.

With the 4-h storage experiments, not only temperatures above 40 °C but also lower temperatures appeared to reduce gametocyte infectivity. It was surprising to find that storing the gametocytes in the thermos flask in water at 37 °C for 4 h was associated with reduced transmission efficiency in some experiments. This was seen most often when the ambient temperature was high (i.e. 32 °C or 42 °C), and not when it was the typical room temperature in an air-conditioned European laboratory (~ 21.3 °C). The temperature of 37 °C would be hypothesized to be ideal for gametocytes, as it mimics their natural environment in the body, and previous studies have indeed shown that *P. falciparum* gametocyte activation in vitro was prevented when they were maintained at 37 °C for 1 h [24]. In agreement with this, short-term storage for 15 min at temperatures up to 40 °C in the study presented here did not reduce transmission. This suggests that either the duration of exposure to high temperatures, or the fact that the gametocytes are stored in venous blood collected in lithium heparin anticoagulant, or both, may also be important factors for gametocyte inactivation, although this is not evaluated here and warrants further investigation. Similarly, the increase in transmission efficiency following 4-h storage compared to immediate feeding, as observed in some experiments, also requires further study. Factors such as the presence of anticoagulants during storage and the duration of storage permitting continued gametocyte maturation may play a role.

Altogether, the data demonstrates that temperature fluctuations influence gametocyte infectivity, seen most acutely with higher temperatures, including high ambient temperatures. Temperature should thus be carefully controlled when collecting and transporting gametocyte infected blood in regions where ambient temperatures could reach over 40 °C. An effect of temperature on gametocyte infectivity has been seen before, however, the current study adds value with the large number of replicates in the SMFA evaluating varying thermos and ambient temperatures. This allowed an informed decision on the optimal temperature conditions for use in DMFA experiments using natural gametocytes carriers in the field.

## Conclusion

This study presents the validation of a method to maintain *P. falciparum* gametocyte infectivity during blood collection and transportation for DMFAs. The optimal conditions (storage in a thermos flask in water at 35.5 °C for up to 4 h with the thermos flask stored between 21.3 and 32 °C) were determined in SMFAs and verified using gametocyte infected venous blood samples from natural gametocyte carriers. With the proposed approach, samples can be transported from more remote settings to the insectary within 4-h without affecting gametocyte infectivity and thus maintaining assay quality. This method will facilitate widespread, accurate assessment of malaria transmission dynamics in the field and this knowledge will contribute to malaria control strategies as progress is made towards elimination.

## Supplementary Information

**Additional file 1: Table S1.** Thermos storage and water temperatures measured during SMFAs.

## Data Availability

The datasets used and/or analysed during the current study are available from the corresponding author on reasonable request.
