# Supplementary material for: Maintaining Plasmodium falciparum gametocyte infectivity during blood collection and transport for mosquito feeding assays in the field
Source: Malar J. 2021 Apr 20;20:191. doi: 10.1186/s12936-021-03725-y (PMC8056727; doi:10.1186/s12936-021-03725-y)
Supplement: Supplementary file 1 — Additional file 1: Table S1. Thermos storage and water temperatures measured during SMFAs. [file 12936_2021_3725_MOESM1_ESM.docx]

**Maintaining *Plasmodium falciparum* gametocyte infectivity during blood collection and transport for mosquito feeding assays in the field**

Harouna M. Soumare^1†^, Wamdaogo Moussa Guelbeogo^2†^, Marga van de Vegte-Bolmer^3^, Geert-Jan van Gemert^3^, Zongo Soumanaba^2^, Alphonse Ouedraogo^2†^, Maurice S. Ouattara^2^, Ahmad Abdullahi^1^, Lamin Jadama^1^, Muhammed M. Camara^1^, Pa Modou Gaye^1^, Michael Mendy^1^, Nwakanma Davis^1^, Alfred B. Tiono^2^, Umberto D’Alessandro^1^, Chris Drakeley^4^, Teun Bousema^3^, Marta Moreno^4#^, Katharine A Collins^3#,*^

^1^Medical Research Council Unit The Gambia at the London School of Hygiene and Tropical Medicine, Banjul, The Gambia

^2^Centre National de Recherche et de Formation sur le Paludisme, Ouagadougou, Burkina Faso

^3^Department of Medical Microbiology, Radboud university medical center, Nijmegen, The Netherlands

^4^Department of Biology of Infection, London School of Hygiene and Tropical Medicine, Faculty of Infectious and Tropical Diseases, London, UK

^†^ These authors contributed equally

^#^ These authors contributed equally

*Corresponding author

Email: Katharine.a.collins@gmail.com

# Additional file 1.

Table S1. Thermos storage and water temperatures measured during SMFAs

| Condition (water@thermos) | Replicate | Thermos storage temp (°C) | Water temperature (°C) | | | |
| --- | --- | --- | --- | --- | --- | --- |
|  |  |  | Start | 2 hours | 4 hours | Change |
| 35.5 @ RT | 1 | NM | 35.4 | 34 | 32.7 | -2.7 |
| 35.5 @ RT | 2 | 22.1 | 35.4 | 33.8 | 32.5 | -2.9 |
| 35.5 @ RT | 3 | 20.4 | 35.5 | 34.2 | 32.9 | -2.6 |
| 37 @ RT | 1 | 21.2 | 37.1 | 35.2 | 33.8 | -3.3 |
| 37 @ RT | 2 | NM | 36.9 | 35.3 | 34.0 | -2.9 |
| 37 @ RT | 3 | 22.1 | 36.9 | 35.1 | 33.8 | -3.1 |
| 37.8 @ RT | 1 | 21.7 | 37.8 | NM | 34.5 | -3.3 |
| 37.8 @ RT | 2 | 20.4 | 37.8 | 35.47 | 34.3 | -3.5 |
| 37.8 @ RT | 3 | 21.4 | 37.8 | 35.8 | 34.4 | -3.4 |
| 35.5 @ 32 | 1 | 31.9 | 35.4 | 34.7 | 34.1 | -1.3 |
| 35.5 @ 32 | 2 | 32.2 | 35.5 | 34.7 | 34.3 | -1.2 |
| 35.5 @ 32 | 3 | 31.9 | 35.5 | 34.9 | 34.5 | -1.0 |
| 37 @ 32 | 1 | 31.9 | 36.9 | 35.9 | 35.4 | -1.5 |
| 37 @ 32 | 2 | 32.2 | 37 | 36.2 | 35.6 | -1.4 |
| 37 @ 32 | 3 | 31.9 | 37 | 35.9 | 35.2 | -1.8 |
| 37.8 @ 32 | 1 | 32.2 | 37.8 | 36.5 | 35.9 | -1.9 |
| 37.8 @ 32 | 2 | 31.9 | 37.8 | 36.6 | 36.0 | -1.8 |
| 37.8 @ 32 | 3 | 31.9 | 37.8 | 36.4 | 35.4 | -2.4 |
| 35.5 @ 42 | 1 | 41.4 | 35.6 | 35.5 | 35.9 | 0.3 |
| 35.5 @ 42 | 2 | 41.5 | 35.4 | 35.5 | 35.8 | 0.4 |
| 35.5 @ 42 | 3 | 42.1 | 35.5 | 35.9 | 36.2 | 0.7 |
| 35.5 @ 42 | 4 | 42.1 | 35.5 | 35.9 | 36.2 | 0.7 |
| 37 @ 42 | 1 | 42 | 37.2 | 36.8 | 36.9 | -0.3 |
| 37 @ 42 | 2 | 41.4 | 36.9 | 36.7 | 36.7 | -0.2 |
| 37 @ 42 | 3 | 41.5 | 36.9 | 36.4 | 36.7 | -0.2 |
| 37.8 @ 42 | 1 | 42 | 37.8 | NM | 37.7 | -0.1 |
| 37.8 @ 42 | 2 | 42.1 | 37.8 | 37.7 | 36.7 | -1.1 |
| 37.8 @ 42 | 3 | 42.1 | 37.8 | 37.8 | 37.9 | 0.1 |
| 37.8 @ 42 | 4 | 42.1 | 37.8 | 37.8 | 37.9 | 0.1 |

NM = not measured
